# Screening Strategies for Silent Myocardial Ischemia in Individuals With Diabetes Without Known Coronary Artery Disease: Systematic Review and Meta‐Analysis

**DOI:** 10.1002/clc.70470

**Published:** 2026-10-05

**Authors:** Vanessa del Carmen Romero Martínez, Juan Pablo Tobón Cuenca, Paula Alejandra Longas García, Luis Eduardo Salazar Moreno, Gloria Ibis Tirado Romero

**Affiliations:** ^1^ Universidad Simón Bolívar Barranquilla Colombia; ^2^ Pontificia Universidad Javeriana Bogotá Colombia; ^3^ Fundación Universitaria Juan N. Corpas Universidad del Tolima Ibagué Colombia; ^4^ Universidad de Ciencias Aplicadas y Ambientales (UDCA) Universidad de Ciencias Aplicadas y Ambientales (UDCA) Bogotá Colombia; ^5^ Fundación Universitaria de Ciencias de la Salud (FUCS) Bogotá Colombia

**Keywords:** coronary artery disease, diabetes mellitus, mass screening, myocardial ischemia, tomography, X‐ray computed

## Abstract

**Introduction:**

Diabetes mellitus markedly increases atherosclerotic cardiovascular risk, and coronary artery disease remains a leading cause of morbidity and mortality. Silent myocardial ischemia (SMI) is clinically relevant in diabetes because autonomic dysfunction and altered pain perception may attenuate typical ischemic symptoms; however, the clinical benefit of routine screening in asymptomatic individuals without known coronary artery disease (CAD) remains uncertain.

**Objective:**

To evaluate whether systematic screening for silent myocardial ischemia or occult CAD in asymptomatic adults with diabetes and no known CAD improves major cardiovascular outcomes.

**Methods:**

We performed a systematic review of studies published from January 1, 2000 through March 31, 2025. The primary effectiveness synthesis included randomized trials comparing systematic screening with no systematic screening or usual care. Primary outcomes were major adverse cardiovascular events (MACE), cardiovascular mortality, and all‐cause mortality; secondary outcomes included detection yield, downstream testing or revascularization, and reported screening‐related harms. Compatible study‐level hazard ratios (HRs) and risk ratios (RRs) were synthesized on the log scale using random‐effects inverse‐variance meta‐analysis, with heterogeneity and sensitivity analyses prespecified. The review was not prospectively registered.

**Results:**

Five RCTs (*n* = 3315; DIAD, DYNAMIT, FACTOR‐64, DADDY‐D, and the pilot trial by Faglia et al.) met the eligibility criteria. Screening did not significantly reduce major cardiovascular outcomes in the four trials included in the quantitative synthesis (pooled HR/RR 0.76; 95% CI 0.48–1.19). DYNAMIT was synthesized narratively because its published primary composite was not directly harmonizable with the pooled endpoint. Reporting of false‐positive results and procedure‐related harms was incomplete and heterogeneous.

**Conclusions:**

In asymptomatic adults with diabetes and no known CAD, routine screening detects occult ischemia or atherosclerosis but has not demonstrated a reduction in MACE or mortality compared with the trial‐era usual‐care or protocol‐defined medical‐management strategies studied. These findings do not support routine population‐wide screening; selective testing is most defensible when the result is expected to change management.

## Graphical abstract

Routine screening for silent myocardial ischemia in asymptomatic adults with diabetes detected occult coronary disease but did not reduce major cardiovascular events or mortality. Current evidence supports intensive cardiovascular risk‐factor management and selective cardiac testing when results are expected to change clinical management.

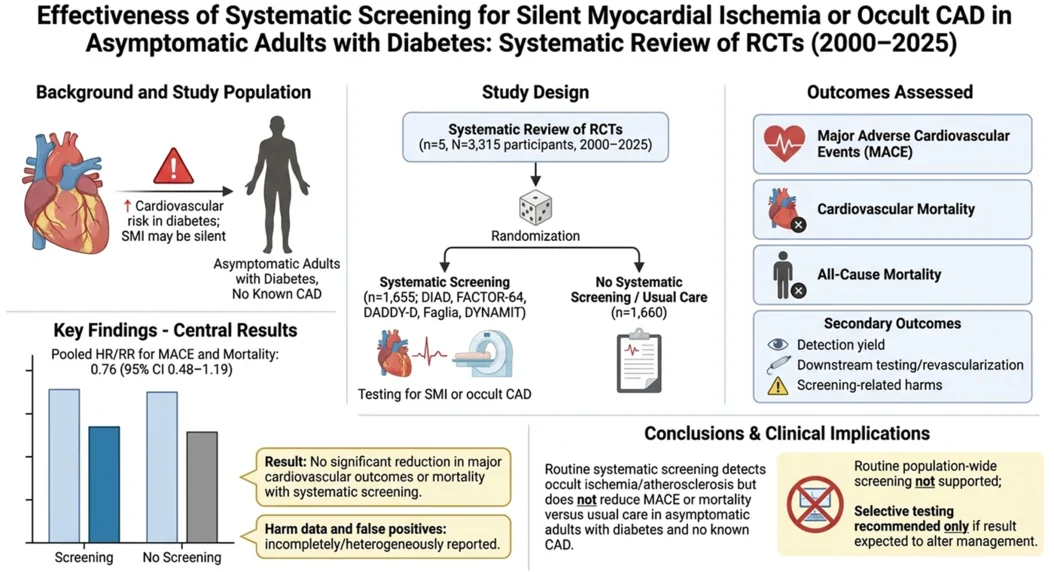

## 1 Introduction

Diabetes mellitus is associated with substantially increased atherosclerotic cardiovascular risk, and coronary artery disease (CAD) remains a major cause of morbidity and mortality in this population [1]. Silent myocardial ischemia (SMI)—objective ischemia without typical angina—is particularly relevant in diabetes because autonomic dysfunction and altered pain perception may blunt warning symptoms. In the baseline Detection of Ischemia in Asymptomatic Diabetics (DIAD) evaluation, approximately 22% of asymptomatic adults with type 2 diabetes had an abnormal screening myocardial perfusion study, while only about 6% had moderate‐to‐large perfusion defects [2]. The availability of exercise electrocardiography, stress echocardiography, radionuclide perfusion imaging, ambulatory ECG, coronary artery calcium (CAC) scoring, and coronary CT angiography (CCTA) has therefore sustained interest in whether earlier detection can improve clinical outcomes. Current guidelines emphasize intensive cardiovascular risk reduction for people with diabetes but do not recommend routine CAD screening in all asymptomatic patients [1, 3].

Randomized trials have not demonstrated that routine screening of asymptomatic adults with diabetes improves hard cardiovascular outcomes; however, the background care against which screening was tested was not uniform. In DIAD, screening results were disclosed, but subsequent investigation and treatment were left to the treating physician rather than prescribed by the trial, and use of preventive medications increased similarly in both groups [4]. DYNAMIT also left post‐screening investigation and treatment to clinical discretion; at study inception, statins were used by only about one third of participants, although approximately 70% received renin–angiotensin system therapy [5]. FACTOR‐64 differed by specifying usual‐care targets for glycated hemoglobin, LDL cholesterol, and systolic blood pressure in both groups and more intensive targets when CCTA detected CAD [6]. DADDY‐D followed participants in diabetes clinics according to contemporaneous ESC guidance, but baseline preventive‐drug use was incomplete and increased during follow‐up [7]. Thus, these trials tested screening on top of different versions of trial‐era usual care rather than a single, uniformly aggressive guideline‐directed medical‐therapy platform.

Several issues nevertheless keep the question clinically relevant. Anatomic measures such as CAC can improve risk stratification in diabetes, while functional imaging can identify higher‐risk ischemic phenotypes [8, 9, 10]. Importantly, the BARDOT program was not part of our primary screening‐versus‐usual‐care evidence set because it did not provide the required unscreened comparator for the effectiveness question. In addition, the 10‐year BARDOT follow‐up was published in July 2025, after our prespecified search cutoff of 31 March 2025; it is mentioned only as post‐search contextual evidence and was not included in study selection or quantitative synthesis [11]. These distinctions are important because prognostic discrimination is not equivalent to proof that a screening strategy improves outcomes.

Because the principal randomized trials were already largely neutral, this review was not designed around a hypothesis that routine screening would improve outcomes. Instead, we sought to determine whether the null findings were consistent across screening modalities and baseline‐risk profiles, and whether their interpretation was influenced by differences in the care actually delivered at baseline and during follow‐up. We therefore examined, where reported, diabetes type, diabetes‐related microvascular complications and other prognostic markers, the intensity and evolution of preventive therapy, downstream angiography/revascularization, and screening‐related harms in addition to test yield and cardiovascular outcomes [4, 5, 6, 7].

Our primary research question was: among asymptomatic adults with diabetes mellitus and no known CAD, does systematic noninvasive screening for silent myocardial ischemia or occult CAD reduce MACE or mortality compared with no systematic screening/usual care? Secondary questions addressed the detection yield of each modality, whether screening changed downstream management, which baseline characteristics were associated with subsequent cardiovascular events, and what screening‐related harms were reported. We specifically sought data on false‐positive testing, invasive angiography, revascularization, and procedure‐related adverse events because screening may generate downstream cascades without improving outcomes [12].

We conducted the review according to PRISMA 2020 and Cochrane methodological guidance [13, 14]. The search covered January 1, 2000 through March 31, 2025. We prespecified clinical outcomes (MACE and cardiovascular/all‐cause mortality), detection yield, downstream management, and reported harms, and planned narrative analyses when outcome definitions or management pathways were too heterogeneous for valid pooling.

## 2 Methods

### 2.1 Study Design

We conducted a systematic review of screening strategies for silent myocardial ischemia or occult CAD in adults with diabetes and no known CAD. Reporting followed PRISMA 2020 and methodological guidance from the Cochrane Handbook [13, 14]. The review was not prospectively registered in PROSPERO or another public registry. This absence of prospective registration is reported transparently and considered a methodological limitation.

### 2.2 Eligibility Criteria

#### 2.2.1 Types of Studies

We included randomized controlled trials (RCTs) and prospective comparative cohorts that compared a systematic screening strategy with no systematic screening or usual care for clinical effectiveness outcomes. Head‐to‐head test‐comparison studies in which all participants underwent a screening modality were not eligible for the primary effectiveness synthesis. We restricted the publication window to January 1, 2000 through March 31, 2025.

#### 2.2.2 Participants

We operationally distinguished two target conditions [1]: silent myocardial ischemia (SMI), defined as inducible ischemia on functional testing in an asymptomatic individual; and [4] significant coronary lesions, defined as anatomic CAD on imaging (e.g., CCTA stenosis or substantial CAC burden). Eligible participants were asymptomatic adults with diabetes and no known CAD. We did not restrict eligibility by diabetes type; type 1 versus type 2 diabetes, diabetes duration, kidney disease/albuminuria, retinopathy, peripheral/autonomic neuropathy, and other reported vascular complications were extracted as potential prognostic or effect‐modifying characteristics. Because functional ischemia and anatomic atherosclerosis are not interchangeable, results were synthesized by target condition and modality when appropriate.

#### 2.2.3 Interventions/Exposures

Eligible screening modalities comprised exercise ECG (treadmill or bicycle), myocardial perfusion imaging (nuclear SPECT or equivalent), pharmacologic or exercise stress echocardiography, ambulatory ECG/Holter, and anatomic imaging with coronary artery calcium (CAC) scoring and/or coronary CT angiography (CCTA).

#### 2.2.4 Outcomes

##### 2.2.4.1 Primary Outcomes

Major adverse cardiovascular events (MACE) as defined by each trial (e.g., cardiac death, non‐fatal myocardial infarction, and unstable angina requiring hospitalization).

Cardiovascular mortality and all‐cause mortality.

##### 2.2.4.2 Secondary Outcomes

Detection yield of previously unrecognized ischemia/CAD (test positives; moderate–large perfusion defects; ≥ 50%–70% stenosis on CCTA/angiography).

Downstream management (additional testing, angiography, revascularization).

Harms of screening (false positives, procedure‐related adverse events).

### 2.3 Search Methods for Identification of Studies

#### 2.3.1 Electronic Searches

We searched MEDLINE (PubMed), Embase, Cochrane CENTRAL, Web of Science Core Collection, and Scopus for records published from January 1, 2000 through March 31, 2025, without language restrictions. We combined controlled vocabulary and keywords for diabetes, asymptomatic/silent ischemia, screening modalities (exercise ECG, stress echocardiography, SPECT/MPI, Holter, CAC, CCTA), and cardiovascular outcomes. Search planning, reference management, and documentation followed Cochrane guidance [14].

Example PubMed strategy (used and adapted for each database):

(Diabetes Mellitus[MeSH] OR diabetes[tiab]) AND (asymptomatic[tiab] OR “silent ischemia”[tiab] OR “inducible ischemia”[tiab]) AND ((electrocardiography, exercise[MeSH] OR “exercise ECG”[tiab] OR treadmill[tiab]) OR (myocardial perfusion imaging[MeSH] OR SPECT[tiab]) OR (“stress echocardiography”[tiab] OR echocardiography, stress[MeSH]) OR (Holter[tiab] OR ambulatory electrocardiography[MeSH]) OR (“coronary artery calcium”[tiab] OR calcium score[tiab] OR coronary calcium[tiab]) OR (coronary CT angiography[tiab] OR angiography, CT, coronary[MeSH] OR CCTA[tiab])) AND (screen*[tiab] OR mass screening[MeSH]).

#### 2.3.2 Other Sources

We searched ClinicalTrials.gov and the WHO ICTRP for ongoing/unpublished studies; screened reference lists and citing articles of included studies and relevant reviews; and hand‐searched major conference proceedings (ACC, AHA, ESC/EASD). We also contacted study authors for clarifications or missing data, and we recorded all search decisions to ensure reproducibility per‐Cochrane guidance.

### 2.4 Data Collection and Analysis

#### 2.4.1 Study Selection

Two reviewers independently screened titles/abstracts and full texts against the prespecified eligibility criteria, with disagreements resolved by consensus or a third reviewer. At title/abstract screening, records were excluded when they clearly failed one or more core eligibility domains (population, screening intervention/comparator, eligible prospective comparative design, or relevant outcomes); reason‐specific counts were not assigned at this stage. Aggregate reasons for exclusion at the full‐text stage are reported in the PRISMA flow diagram [13].

#### 2.4.2 Data Extraction and Management

We used standardized, piloted extraction forms to capture study design, diabetes type, baseline cardiovascular and diabetes‐related risk characteristics, screening modality, comparator, follow‐up, clinical outcomes, and effect measures. To address the clinical context of each trial, we additionally extracted—where reported—baseline and achieved preventive therapy (including lipid‐lowering, renin–angiotensin system therapy, antiplatelet therapy, blood‐pressure/glycemic targets), protocol rules for post‐test management, downstream testing, angiography, revascularization, and screening‐related adverse events. Prognostic factors such as albuminuria/CKD, autonomic or peripheral neuropathy, peripheral vascular disease, and imaging severity were also recorded. Two reviewers extracted data independently; a third checked for consistency.

### 2.5 Risk‐of‐Bias (Critical Appraisal)

We appraised RCTs with RoB 2 across the randomization process, deviations from intended interventions, missing outcome data, outcome measurement, and selection of the reported result. Two reviewers assessed risk of bias independently, with consensus adjudication. Risk‐of‐bias judgments were incorporated into interpretation and sensitivity analyses in accordance with Cochrane guidance [14].

### 2.6 Bias Control Procedures

When outcome data were missing or incompletely reported, we contacted authors. If unrecoverable, we used available‐case analyses and explored impacts in sensitivity analyses (e.g., best‐/worst‐case bounds where appropriate), consistent with Cochrane recommendations on incomplete outcome data and missing data.

### 2.7 Synthesis Plan (Quantitative and Qualitative)

Effect measures: For time‐to‐event outcomes (e.g., MACE, mortality) we pooled log‐HRs using inverse‐variance weighting. For dichotomous outcomes (e.g., detection yield, revascularization) we pooled RRs. When event rates were low, we accepted HRs and RRs as approximately comparable and analyzed on the log scale.

Model: We prespecified random‐effects meta‐analysis (DerSimonian–Laird) with inverse‐variance weighting; we reported τ^2^ and I^2^ to quantify heterogeneity and explored clinical/methodological heterogeneity as per Cochrane guidance.

Sensitivity analyses: We repeated meta‐analyses excluding studies at high overall risk of bias; using fixed‐effect models; applying alternative continuity corrections for zero‐event cells; and removing outliers (leave‐one‐out).

We quantified between‐study heterogeneity with Cochran's Q, τ^2^ (DerSimonian–Laird), and I^2^, and reported a 95% prediction interval for the pooled effect. Sensitivity analyses included (i) model specification checks (fixed‐effect and Hartung–Knapp–Sidik–Jonkman random‐effects), (ii) leave‐one‐out meta‐analysis, and (iii) small‐study effects assessment by funnel‐plot inspection and Egger's regression (with Begg–Mazumdar rank correlation as a robustness check), acknowledging limited power with ≤ 10 studies. Two‐sided α = 0.05 was used for hypothesis testing.

Narrative synthesis: Where pooling was inappropriate (few studies, incompatible definitions), we synthesized findings descriptively, emphasizing direction and consistency of effects and precision.

## 3 Results

### 3.1 Study Selection and Characteristics

Five randomized trials met the primary effectiveness eligibility criteria (*n* = 3315): four full‐scale RCTs and one open‐label pilot RCT (Figure 1). All compared a systematic screening strategy with no systematic screening/usual care. The included modalities were exercise ECG, stress echocardiography, myocardial perfusion imaging, and CCTA. The evidence base predominantly comprised type 2 diabetes.

- Faglia et al. (2005)—Open‐label pilot RCT in 141 high‐cardiovascular‐risk adults with type 2 diabetes; 71 were assigned to screening with exercise ECG plus dipyridamole stress echocardiography, followed by angiography/revascularization when indicated [15].
- DIAD (2009, USA/Canada)—Multicenter RCT of 1123 adults with type 2 diabetes, randomized to adenosine stress MPI (SPECT) screening or no screening; downstream management was left to treating physicians [4].
- DYNAMIT (2011, France)—Multicenter RCT of 631 high‐risk adults with type 2 diabetes, randomized to systematic ischemia screening (exercise testing with SPECT when needed) or usual care; recruitment stopped early because of slow enrollment and lower‐than‐expected event rates [5].
- FACTOR‐64 (2014, USA)—Multicenter RCT of 900 adults with type 1 or type 2 diabetes, randomized to CCTA‐guided screening or standard national‐guideline‐based diabetes care; the protocol specified preventive‐treatment targets [6].
- DADDY‐D (2015, Italy)—Single‐center RCT of 520 high‐risk adults with type 2 diabetes, randomized to exercise ECG screening with angiography/revascularization when indicated or regular follow‐up [7].

**FIGURE 1:**
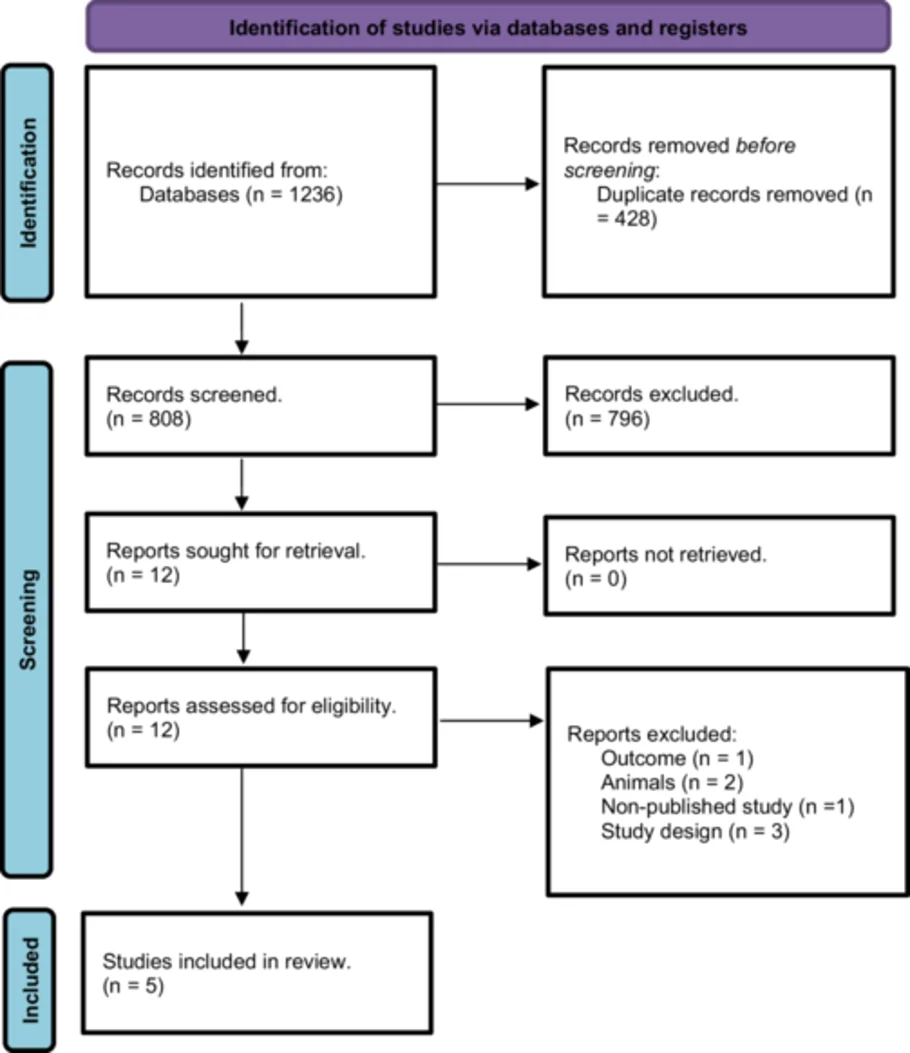
PRISMA 2020 flow diagram of study selection.

Of 808 unique records screened by title/abstract, 796 were excluded because they did not meet one or more prespecified eligibility domains (e.g., ineligible population, no eligible systematic screening comparator, ineligible study design, or no relevant clinical outcomes). Reason‐specific counts were not coded at the title/abstract stage. Twelve reports underwent full‐text assessment; seven were excluded for the reasons displayed in Figure 1 (outcome, *n* = 1; animal study, *n* = 2; non‐published report, *n* = 1; ineligible study design, *n* = 3), leaving five RCTs. The head‐to‐head modality trial previously represented in Table 1 as “Barthélémy 2010” was the Jacqueminet et al. randomized comparison of SPECT versus stress echocardiography; because it lacked a no‐screen/usual‐care comparator, it was not eligible for the primary effectiveness synthesis and has been removed from the included‐study table [16].

**TABLE 1:** Summarizes screening yield and immediate downstream invasive management in the five included trials.

Study (Year)	Modality	Screened sample	Yield	Downstream procedures
Faglia et al., 2005	Exercise ECG ± stress echo	71 (T2D; very high risk)	≈21% positive	Angiography for all positives; 11 PCI/CABG
DIAD, 2009	Adenosine SPECT MPI	561 assigned; 522 interpretable (T2D)	113/522 (22%) abnormal; ≈6% moderate–large	5.5% revascularized during follow‐up
DYNAMIT, 2011	Exercise test ± SPECT	316 (T2D; high risk)	68/316 (21.5%) positive/uncertain	38 angiography; 12 PCI/CABG
FACTOR‐64, 2014	CCTA + CAC	452 (T1D/T2D)	≈23% ≥moderate anatomic CAD	26 revascularizations reported
DADDY‐D, 2015	Exercise ECG (ETT)	262 (T2D; high risk)	20/262 (7.6%) positive	17 angiography; 12 revascularized

*Note:* Screening yield and downstream invasive management in the five included randomized trials.

Abbreviations: SMI, silent myocardial ischemia; MPI, myocardial perfusion imaging; ETT, exercise tolerance test; CCTA, coronary CT angiography; PCI, percutaneous coronary intervention; CABG, coronary artery bypass grafting.

### 3.2 Background Preventive Care, Diabetes Phenotype, and Prognostic Factors

The intensity and structure of preventive care differed materially across trials. DIAD did not prescribe post‐screening therapy; subsequent management was at the treating physician's discretion, although statin, ACE‐inhibitor, and aspirin use increased over follow‐up in both groups [4]. DYNAMIT described participants as receiving appropriate care for the era, but statins were used by only about one third at study inception and approximately 70% received renin–angiotensin system therapy; discharge treatments were similar between groups except for a trend toward more antiplatelet use after screening [5]. FACTOR‐64 had the most explicit management protocol, with standard‐care targets of HbA1c < 7.0%, LDL‐C < 100 mg/dL, and systolic blood pressure < 130 mmHg in both groups and more intensive targets after CCTA‐detected CAD [6]. In DADDY‐D, participants were followed in diabetes clinics according to contemporaneous ESC guidance; statin use increased from 49.2% to 63.7% in the screened group and from 44.2% to 58.9% in controls, illustrating that baseline therapy was not uniformly intensive [7].

Four trials enrolled type 2 diabetes exclusively; FACTOR‐64 was the only included trial enrolling both type 1 and type 2 diabetes, with type 1 diabetes representing only a minority of participants, which limits inference for type 1 diabetes [6]. Prognostic analyses also suggested that baseline diabetes‐related complications could be more informative for event risk than screening assignment. In DIAD, male sex, longer diabetes duration, microalbuminuria/proteinuria, renal dysfunction, peripheral neuropathic symptoms or sensory loss, cardiac autonomic dysfunction, peripheral vascular disease, elevated LDL‐C, and family history of premature CAD were associated with the primary cardiac endpoint; moderate‐to‐large MPI defects were also prognostic [4, 17]. In DYNAMIT, increased urinary albumin excretion was the only adjustment variable with significant independent prognostic value (HR 2.58; 95% CI 1.45–4.58) [5]. These findings support risk enrichment based on clinical and microvascular phenotype but do not establish that screening improves outcomes within those subgroups.

### 3.3 Detection of Silent Coronary Artery Disease

Screening strategies did identify a proportion of asymptomatic diabetic patients with silent ischemia or previously unrecognized CAD. However, the prevalence of abnormal screening tests varied by modality and population risk, generally ranging from about 8% up to 22% (with higher yields in higher‐risk groups):

- Myocardial perfusion imaging (MPI): In DIAD, 113 of 522 participants with interpretable screening studies (22%) had an abnormal stress MPI; only about 6% had moderate‐to‐large perfusion defects [2, 4]. The finding confirmed that SMI was detectable in a clinically meaningful minority, but the randomized comparison did not show improved outcomes from assigning patients to screening.
- Multistep exercise/SPECT screening: In DYNAMIT, 68 of 316 screened participants (21.5%) had a positive or uncertain initial screening result. Thirty‐eight (12.0%) underwent coronary angiography, followed by PCI in nine and CABG in three [5].
- Exercise ECG stress testing: In DADDY‐D, 20 of 262 screened participants (7.6%) had a positive exercise tolerance test. Seventeen proceeded to coronary angiography and 12 (4.6% of the screened group) underwent revascularization [7].
- Coronary CT angiography: In FACTOR‐64, CCTA identified some degree of coronary atherosclerosis in most screened participants. Approximately 23% had at least moderate anatomic disease (including moderate or severe stenosis), whereas nonobstructive plaque was common [6]. This high anatomic yield did not translate into a statistically significant reduction in clinical events.

### 3.4 Downstream Management and Screening‐Related Harms

Reporting of screening‐related harms was sparse and heterogeneous, precluding pooled estimates of false‐positive results or procedure‐related adverse events. As summarized in Table 1, systematic screening led to downstream angiography and revascularization in only a minority of participants. DYNAMIT reported no serious adverse events during the initial screening hospitalization, although adverse events during subsequent follow‐up were not systematically recorded [5]. Across trials, no uniform reference‐standard definition of a false‐positive screening result was available. Therefore, screening‐related harms could be described only narratively.

### 3.5 Major Cardiovascular Events

None of the larger randomized trials demonstrated a statistically significant reduction in major cardiovascular events with routine screening. The small Faglia pilot was the exception, but its open‐label design, small sample, and broader event definition make it less generalizable. Trial‐specific findings are summarized below.

- DIAD [4]: After approximately 4.8 years, cardiac death or nonfatal MI occurred in 15 screened participants versus 17 controls (HR 0.88; 95% CI 0.44–1.88; *p* = 0.73). Moderate‐to‐large perfusion defects were prognostic, but DIAD was not designed to test a specific treatment strategy after a positive scan: downstream investigation and therapy were left to clinicians, and few participants underwent early angiography/revascularization. The trial therefore supports a neutral screening‐strategy result but does not establish that a protocolized intervention for screen‐positive patients is ineffective.
- DADDY‐D [7]: Over a median 3.6 years, the first cardiac event (cardiac death or nonfatal MI) occurred in 12/262 screened participants (4.6%) versus 14/258 controls (5.4%; HR 0.85; 95% CI 0.39–1.83; *p* = 0.68). Heart‐failure events were also not significantly different. Subgroup signals in older participants were exploratory and should not be interpreted as definitive evidence of benefit.
- FACTOR‐64 [6]: At 4 years, the primary composite of all‐cause mortality, nonfatal MI, or unstable‐angina hospitalization occurred in 6.2% of the CCTA‐screened group versus 7.6% of controls (HR 0.80; 95% CI 0.49–1.32; *p* = 0.38). The trial used explicit preventive‐treatment targets, but between‐group differences in achieved risk‐factor control were modest; CCTA‐guided screening did not significantly reduce clinical events.
- DYNAMIT [5]: The trial stopped early because of recruitment difficulties and lower‐than‐expected event rates. Twenty‐eight participants in the screening group and 26 controls experienced the broad primary composite (all‐cause death, nonfatal MI, nonfatal stroke, or heart failure requiring hospital/emergency intervention), yielding an adjusted HR of 1.00 (95% CI 0.59–1.71). Importantly, preventive care was not equivalent to current practice: statins were used by only about one third of participants at study inception.
- Faglia et al. (2005) [15]: This open‐label pilot RCT suggested fewer combined cardiac events after screening and selective revascularization (four events in the screened arm vs. 15 in controls; *p* = 0.018). However, the trial was small, at high risk of bias, and included angina in the composite; the finding was not replicated in the larger trials (Table 2).

**TABLE 2:** Summarizes the major cardiovascular event outcomes from the key trials.

Study (Year)	Outcome	Follow‐up	Events: screening versus control	Effect size
DYNAMIT, 2011	Death, nonfatal MI/stroke, or HF requiring hospital/emergency care	3.5 y	28 versus 26	HR 1.00 (95% CI 0.59–1.71)
Faglia et al., 2005	MI or angina	4.5 y	4 versus 15	RR 0.27; *p* = 0.018
DIAD, 2009	Cardiac death or nonfatal MI	4.8 y	15 (2.7%) versus 17 (3.0%)	HR 0.88 (95% CI 0.44–1.88); *p* = 0.73
DADDY‐D, 2015	Cardiac death or nonfatal MI	3.6 y	12 (4.6%) versus 14 (5.4%)	HR 0.85 (95% CI 0.39–1.83); *p* = 0.68
FACTOR‐64, 2014	Death, MI, or unstable angina	4.0 y	28 (6.2%) versus 34 (7.6%)	HR 0.80 (95% CI 0.49–1.32); *p* = 0.38

*Note:* Major cardiovascular outcomes in the five included randomized trials. The larger trials did not show a statistically significant reduction in clinical events with screening. The Faglia pilot reported benefit for a broader composite of myocardial infarction or angina but was small and at high risk of bias. For Faglia et al., this broader composite is presented descriptively in Table 2, whereas myocardial infarction alone (one event in the screened group vs. 11 in the control group) was used in the quantitative synthesis to improve comparability with the harder cardiovascular endpoints of the other pooled trials.

No consistent reduction in nonfatal MI was demonstrated across the randomized evidence. Point estimates in several trials favored screening, but confidence intervals were wide and crossed the null. The generally low event rates, differences in outcome definitions, and heterogeneity in trial‐era preventive management reduce precision and limit claims of equivalence; the data show no demonstrated benefit rather than proof that screening can never be beneficial in a selected subgroup.

### 3.6 Mortality Outcomes

Across DIAD, DADDY‐D, FACTOR‐64, and DYNAMIT, mortality was uncommon and no trial demonstrated a statistically significant mortality reduction attributable to routine screening. DADDY‐D reported an exploratory signal among older participants, but the subgroup analysis was not prespecified evidence of a treatment effect. The mortality data therefore support the same overall interpretation as the composite cardiovascular outcomes: no demonstrated survival benefit from systematic screening.

Because mortality definitions and reporting differed across trials, and some publications did not provide arm‐specific cause‐specific death counts in a form suitable for harmonization, we did not pool mortality separately or present estimated arm‐specific counts.

### 3.7 Data for Meta‐Analysis (Study‐Level Outcomes)

A meta‐analysis of four randomized trials (Faglia et al., DIAD, DADDY‐D, and FACTOR‐64) with sufficiently harmonized study‐level effect estimates for the prespecified pooled major cardiovascular outcome demonstrated no statistically significant reduction with screening. DYNAMIT was retained in the qualitative synthesis because its published primary HR referred to a broader composite that included all‐cause death, stroke, and heart‐failure emergency care. For Faglia et al., the quantitative synthesis used myocardial infarction alone (one event in the screened group vs. 11 in the control group; RR approximately 0.09), rather than the broader composite of myocardial infarction or angina reported descriptively in Table 2, to improve comparability with the harder cardiovascular endpoints used in the other pooled trials. Using a random‐effects DerSimonian–Laird model for the four harmonized trials, the pooled hazard/risk ratio was 0.76 (95% CI 0.48–1.19), with low‐to‐moderate heterogeneity (I^2^ = 27.4%). The corresponding forest plot is shown in Figure 2.

**FIGURE 2:**
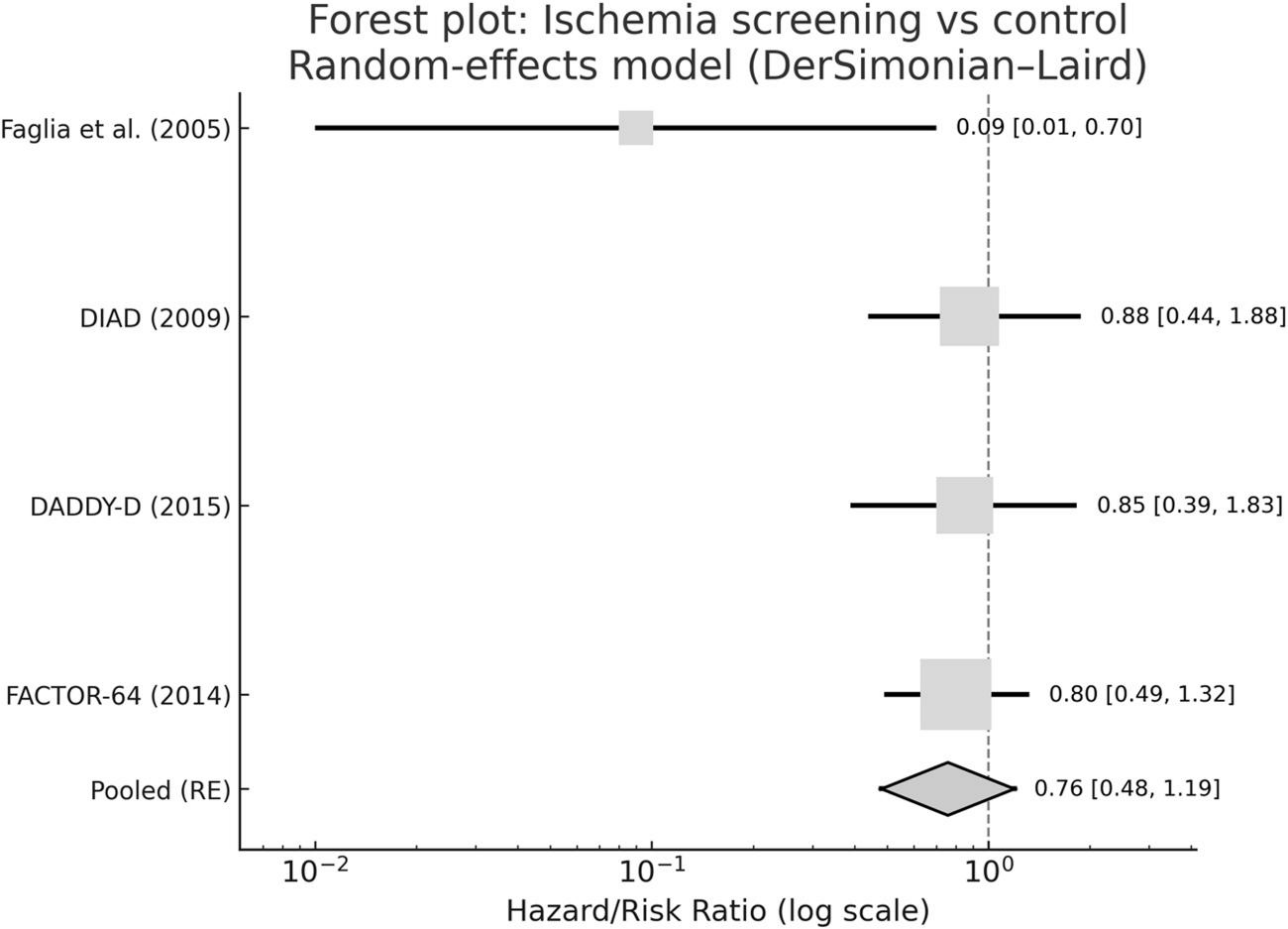
Forest plot of screening versus usual care for major cardiovascular outcomes (random‐effects DerSimonian–Laird model).

### 3.8 Sensitivity Analyses

Between‐study heterogeneity was low‐to‐moderate: Q = 4.13, I^2^ = 27.4%, τ^2^ = 0.060. The random‐effects pooled HR/RR was 0.76 (95% CI, 0.48–1.19), with a 95% prediction interval of 0.39–1.47, indicating that future trial effects are expected to vary around the null. Results were stable across model specifications: the fixed‐effect pooled HR/RR was 0.78 (95% CI, 0.55–1.11), and the Hartung–Knapp pooled HR/RR was 0.76 (95% CI, 0.33–1.75). Leave‐one‐out analyses yielded pooled HR/RRs ranging from 0.65 to 0.83; notably, excluding Faglia et al. reduced heterogeneity to I^2^ = 0%, with a pooled HR/RR of 0.83 (95% CI, 0.58–1.19), whereas excluding any other single trial yielded pooled HR/RRs ranging from 0.65 to 0.68, with I^2^ = 50%. Funnel‐plot inspection did not suggest asymmetry; Egger's intercept was −2.19 (*p* = 0.16) and Begg's τ was −0.33 (*p* = 0.75), providing no statistical evidence of small‐study effects, although these analyses had limited power with only four studies (Figure 3).

**FIGURE 3:**
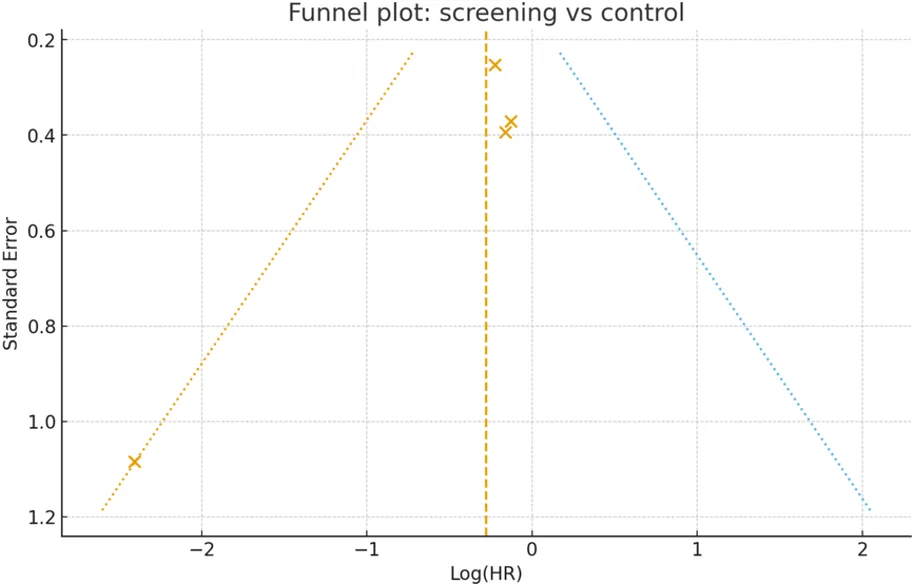
Funnel plot screening versus control.

### 3.9 Risk of Bias Assessment

RoB 2 identified high overall risk of bias for the small Faglia pilot and “some concerns” for DIAD, DYNAMIT, FACTOR‐64, and DADDY‐D (Table 3). Relevant issues included the open‐label nature of screening‐strategy trials, deviations or non‐protocolized downstream management, early stopping and limited power in DYNAMIT, and some concerns regarding selection of reported results. These issues are particularly important because the intervention being tested was a care strategy, not merely the diagnostic performance of a test.

**TABLE 3:** RoB 2 assessment of the five randomized trials of routine CAD screening in asymptomatic diabetes.

Domain	Faglia 2005	DIAD 2009	DYNAMIT 2011	FACTOR‐64 2014	DADDY‐D 2015
Domain 1: Randomization process	Low	Low	Some concerns	Some concerns	Low
Domain 2: Deviations from intended interventions	High	Low	Low	Low	Low
Domain 3: Missing outcome data	High	Low	Low	Low	Low
Domain 4: Measurement of the outcome	High	Low	Low	Low	Some concerns
Domain 5: Selection of the reported result	Some concerns	Some concerns	Some concerns	Some concerns	Some concerns
Overall risk of bias	High	Some concerns	Some concerns	Some concerns	Some concerns

Accordingly, the pooled neutral estimate should be interpreted as an absence of demonstrated clinical benefit, not as proof of equivalence between screening and no screening. Sensitivity analysis excluding the high‐risk Faglia pilot yielded a pooled HR/RR of 0.83 (95% CI, 0.58–1.19), with I^2^ = 0%, supporting the direction of the main finding while retaining substantial imprecision. Risk of bias, low event rates, and heterogeneous post‐test management therefore temper certainty about small benefits or harms in narrowly defined high‐risk subgroups.

## 4 Discussion

This review evaluated whether systematic screening for silent myocardial ischemia or occult CAD improves outcomes in asymptomatic adults with diabetes. Across the randomized evidence, routine screening did not demonstrate lower rates of major cardiovascular events or mortality compared with the care delivered in each trial. The comparator, however, should not be described as a uniform guideline‐directed medical‐therapy platform: post‐test care was physician‐directed in DIAD, background prevention was incompletely implemented in DYNAMIT, DADDY‐D intensified therapy over follow‐up, and FACTOR‐64 used explicit protocol targets [4, 5, 6, 7]. The neutral effect estimates therefore apply to these heterogeneous trial‐era management pathways rather than to one standardized prevention regimen.

The historical context is central to interpretation. Most participants were enrolled before the current era of routine cardioprotective glucose‐lowering therapy, and the included screening trials did not test strategies layered on contemporary use of SGLT2 inhibitors, GLP‐1 receptor agonists, newer lipid‐lowering options, and current cardiorenal risk‐based treatment algorithms. Even within the older trials, baseline statin and other preventive‐drug use varied substantially. It is therefore not appropriate to characterize prevention in the 2000s and early‐to‐mid 2010s as equivalent to current care. Current cardiometabolic therapy may further reduce event rates and raise the threshold for screening to add incremental benefit, but this is an inference: no included RCT directly tested routine screening on top of today's full preventive armamentarium [18, 19].

A central distinction is between prognostic information and therapeutic benefit. The main BARDOT study, which was not eligible for our screening‐versus‐usual‐care comparison, showed that abnormal perfusion imaging identifies higher‐risk patients [10, 20]. Its 10‐year follow‐up provides additional prognostic context but was published in July 2025, after our 31 March 2025 search cutoff, and therefore was not part of the systematic‐review evidence set [11]. These data support the prognostic value of imaging but do not establish that screening itself improves hard outcomes.

Anatomic atherosclerosis measures, especially CAC, may still have value for risk stratification. MESA analyses show substantial heterogeneity of cardiovascular risk among people with diabetes according to CAC burden, including lower short‐term risk when CAC = 0 and markedly higher risk with increasing CAC [8, 9]. This supports using CAC selectively to refine prevention decisions, but risk reclassification should not be conflated with evidence that a population screening program reduces events.

Related randomized evidence from stable ischemic heart disease is directionally consistent. In the prespecified diabetes analysis of the ISCHEMIA trials, participants with diabetes had higher absolute risk, but diabetes status did not identify a subgroup with a differential reduction in death or MI from an initial invasive strategy compared with a conservative strategy [21]. Although ISCHEMIA did not study asymptomatic screening and therefore cannot answer our primary question, it reinforces the principle that identifying substantial ischemia does not automatically create an outcome‐improving pathway to revascularization.

These findings are consistent with guidelines that prioritize intensive risk‐factor management and discourage routine screening of all asymptomatic adults with diabetes, while allowing testing for specific clinical questions or selected very‐high‐risk phenotypes [1, 3, 12, 22]. Our review does not prove that every high‐risk subgroup derives no benefit; rather, the available trials have not demonstrated an outcome benefit, and evidence is particularly sparse for type 1 diabetes and for phenotypes defined by severe CKD/albuminuria, autonomic neuropathy, or other microvascular complications.

Modern CCTA protocols reduce radiation exposure compared with earlier generations, which may lower one component of screening harm; however, this technical progress does not resolve the key question of actionability. FACTOR‐64 detected extensive anatomic disease but did not demonstrate fewer clinical events. Radiation‐reduction advances published after our search cutoff are therefore relevant to current implementation but do not alter the randomized effectiveness evidence synthesized here [6, 23].

Several limitations constrain interpretation. First, the review was not prospectively registered in PROSPERO, which limits external verification that all analytic decisions preceded inspection of study results. Second, only five eligible randomized trials were identified, several were underpowered because event rates were lower than expected, and trial populations and screening pathways were heterogeneous. Third, reason‐specific counts were not captured for the 796 title/abstract exclusions, and the individual report‐level exclusion log for the seven full‐text exclusions was not available during revision. Although aggregate full‐text exclusion categories were retained and are reported in the PRISMA diagram, the individual excluded reports required for complete report‐level fulfillment of PRISMA item 16b could not be reliably reconstructed retrospectively. Fourth, background preventive care varied substantially across trials and was not equivalent to current 2025–2026 cardiometabolic practice. Fifth, reporting of false positives, procedure‐related complications, anxiety/cost, and other screening harms was insufficient for quantitative synthesis. Finally, type 1 diabetes, women, advanced CKD, and other high‐risk microvascular phenotypes were underrepresented. Strengths include duplicate screening/extraction, explicit characterization of trial‐era care, RoB 2 assessment, sensitivity analyses, and separation of prognostic test yield from evidence of clinical effectiveness.

For current practice, the most evidence‐based default remains comprehensive cardiovascular risk reduction irrespective of screening status: intensive LDL‐C lowering as indicated, blood‐pressure control, smoking cessation, weight management, and glucose‐lowering therapies with established cardiovascular and kidney benefit. SGLT2 inhibitors and GLP‐1 receptor agonists now form part of this modern prevention landscape [18, 19]. These therapies were not routinely available during enrollment of the screening RCTs, so their presence should be viewed as current clinical context rather than an explanation for the original trial results. Any new screening strategy should demonstrate incremental management changes and clinical benefit beyond this current standard.

A practical interpretation is to distinguish selective risk refinement from routine ischemia screening. CAC can refine estimated atherosclerotic burden and may support decisions about prevention intensity, but it should not replace therapies already indicated by diabetes and global cardiovascular risk. Functional or anatomic testing is better reserved for symptoms, diagnostic uncertainty, or carefully selected high‐risk situations in which the result is expected to change management [1, 8, 9, 22].

Future trials should avoid repeating broadly defined screening strategies without a prespecified action pathway. High‐risk phenotypes—such as marked albuminuria/CKD, severe CAC burden, autonomic dysfunction, or other evidence of diffuse vascular disease—should be prospectively defined, and type 1 and type 2 diabetes should be reported separately. Trials should mandate contemporary background therapy in both groups, specify exactly how screening results alter treatment, and systematically capture false positives, invasive procedures, procedure complications, anxiety, cost, and patient‐reported outcomes. This design would test whether imaging‐guided management adds value beyond modern risk‐guided prevention rather than merely whether a test detects disease.

In conclusion, randomized evidence does not demonstrate that routine screening of asymptomatic adults with diabetes for silent myocardial ischemia or occult CAD reduces major cardiovascular events or mortality compared with the trial‐era usual‐care or protocol‐defined medical‐management strategies studied. Screening can provide prognostic information, but prognostic separation alone is insufficient to justify routine testing without an action pathway that improves outcomes. The evidence is dominated by type 2 diabetes, background preventive therapy varied across trials, and screening‐related harms were incompletely reported. Current practice should prioritize modern multifactorial cardiovascular prevention and use imaging selectively when the result is likely to change management.

## Author Contributions

**Vanessa del Carmen Romero Martínez:** conceptualization, methodology, investigation, data curation, writing – original draft, writing – review and editing. **Juan Pablo Tobón Cuenca:** methodology, validation, data curation, writing – review and editing. **Paula Alejandra Longas García:** investigation, data curation, methodology, writing – original draft, writing – review and editing. **Luis Eduardo Salazar Moreno:** investigation, data curation, formal analysis, writing – original draft, writing – review and editing. **Gloria Ibis Tirado Romero:** conceptualization, methodology, supervision, project administration, writing – original draft, writing – review and editing. All authors reviewed and approved the final version of the manuscript.

## Funding

The authors have nothing to report.

## Ethics Statement

This study is a systematic review and meta‐analysis of published data and did not require ethics committee approval. No identifiable patient data were used.

## Conflicts of Interest

The authors declare no conflicts of interest.

## Data Availability

The data that support the findings of this study are available from the corresponding author upon reasonable request. The data supporting the findings of this study are derived from published studies included in the systematic review and are available within the article and its supplementary materials. Additional extraction files and analytic code are available from the corresponding author upon reasonable request.
